# Electrocardiogram-Based Emotion Recognition Systems and Their Applications in Healthcare—A Review

**DOI:** 10.3390/s21155015

**Published:** 2021-07-23

**Authors:** Muhammad Anas Hasnul, Nor Azlina Ab. Aziz, Salem Alelyani, Mohamed Mohana, Azlan Abd. Aziz

**Affiliations:** 1 Faculty of Engineering and Technology, Multimedia University, Melaka 75450, Malaysia; 1141126389@student.mmu.edu.my (M.A.H.); azlan.abdaziz@mmu.edu.my (A.A.A.); 2 Center for Artificial Intelligence (CAI), King Khalid University, Abha 61421, Saudi Arabia; s.alelyani@kku.edu.sa (S.A.); mmuhanna@kku.edu.sa (M.M.); 3 College of Computer Science, King Khalid University, Abha 61421, Saudi Arabia

**Keywords:** electrocardiogram (ECG), affective computing, emotion recognition system, healthcare

## Abstract

Affective computing is a field of study that integrates human affects and emotions with artificial intelligence into systems or devices. A system or device with affective computing is beneficial for the mental health and wellbeing of individuals that are stressed, anguished, or depressed. Emotion recognition systems are an important technology that enables affective computing. Currently, there are a lot of ways to build an emotion recognition system using various techniques and algorithms. This review paper focuses on emotion recognition research that adopted electrocardiograms (ECGs) as a unimodal approach as well as part of a multimodal approach for emotion recognition systems. Critical observations of data collection, pre-processing, feature extraction, feature selection and dimensionality reduction, classification, and validation are conducted. This paper also highlights the architectures with accuracy of above 90%. The available ECG-inclusive affective databases are also reviewed, and a popularity analysis is presented. Additionally, the benefit of emotion recognition systems towards healthcare systems is also reviewed here. Based on the literature reviewed, a thorough discussion on the subject matter and future works is suggested and concluded. The findings presented here are beneficial for prospective researchers to look into the summary of previous works conducted in the field of ECG-based emotion recognition systems, and for identifying gaps in the area, as well as in developing and designing future applications of emotion recognition systems, especially in improving healthcare.

## 1. Introduction

Research interest in affective computing via physiological modalities has been popularized by the accelerated development of technological solutions, particularly within the healthcare industry. The field of affective computing originated from a paper written by Rosalind Picard in 1995, discussing neurological studies of human emotions and the possibility for computers to mimic them by expression recognition [1]. Affective computing is a multidisciplinary study that revolves around computer science, psychology, cognition, and physiology [2].

The significance of emotions in natural human interaction was demonstrated by Ekman et al. [3] from the premise “If B perceives A’s facial expression of emotion, B’s behavior toward A may change, and A’s noticing this may influence or determine A’s experience of emotion”. Meanwhile, in a book by Reeves et al. [4], the authors claimed that humans treated computers as if they are just another living being too. From both arguments, it can be deduced that if computer systems are capable of discerning and responding to human affects, then the interactional gap between people and machines will be as naturalistic as talking to a friend and improve the human–computer interaction. Adopting emotion recognition systems should be considered as a footstep towards instilling empathy, sympathy, and compassion into artificially intelligent machinery.

Emotion recognition systems have a lot of prospective applications, spanning healthcare, entertainment, e-learning, marketing, human monitoring, and security. According to [5], there were three major applications of emotion recognition systems specifically using ECG signals:

- Firstly, monitoring human emotions during certain tasks and assessing the behavioral response in critical situations. For example, in [6], the emotion recognition system focuses on studying a driver’s performance during a race.
- Next, clinical application in monitoring patients’ psychological condition for relevant drug prescriptions or treatment. In [7], emotion recognition is implemented in healthcare settings to promote relaxation and reduce stress. Three emotional services are provided in the design framework, which are relaxation, amusement, and excitement services.
- Finally, emotion recognition can be used for marketing. Emotion recognition can be utilized for website optimization [8], where the system can be designed to collect information on which adverts attract the most attention, which can allow catering appropriate contents according to audience demography.

The physiological approach towards emotion recognition has become a better alternative to facial expressions, gestures, and vocal traits. Machine vision-based emotion recognition systems are prone to fake emotions and can be manipulated easily [9,10,11]. This is why many studies focused on physiological signals, including the multimodal approach, by combining different physiological signals from biosensors such as an ECG, an electroencephalogram (EEG), an electromyogram (EMG), electrodermal activity (EDA) or galvanic skin response (GSR), a photoplethysmogram (PPG) or blood volume pressure (BVP), or a respiratory inductive plethysmograph (RIP). Although the multimodal emotion recognition approach commonly performed better, the unimodal approach has the advantages of a lower processing time and simpler data collection [12].

The brain and heart are connected via the autonomic nervous system (ANS), in which both indirectly influence each other’s behavior [13]. The connection of the sympathetic nervous system (SNS) and parasympathetic nervous system (PNS) is part of the ANS. Thus, emotional experience does cause some changes in the heart rhythm, and this can be detected through ECG readings. The purpose of this review is to sum up the literature to date that has reported the adoption of ECG as an input of emotion recognition systems. This paper also discusses ECG features such as the heart rate (HR), as well as heart rate variability (HRV), and their relationship with the autonomic innervation of the heart.

The next sections discuss the review methodology, followed by the theoretical background of the autonomic innervation of the heart, electrocardiograms, various emotional models, and emotion elicitation and emotion evaluation techniques. ECG-inclusive datasets are reviewed and analyzed in Section 4. Section 5 discusses the methodology of developing an emotion recognition system from the pre-processing of ECG signals, feature extraction, feature selection and dimensionality reduction, classification, and validation. Section 6 focuses on the discussion of the summarized literature. The applications of emotion recognition systems in healthcare are reviewed in Section 7, and the discussion of the reviews is presented in Section 8. The last section concludes the work.

## 2. Review Methodology

The journals and articles reviewed in this work underwent a thorough selection process. Initially, keywords for the search criteria were identified. Studies associated with “*emotion recognition*”, “*ECG*”, and “*healthcare*” were searched throughout different academic databases. Table 1 shows the publisher database and number of studies reviewed for ECG-based emotion recognition, and healthcare applications of emotion recognition systems. Here, IEEE Xplore was the database with the most papers reviewed.

The exclusion criteria after the first reading included the removal of duplicated publications, contextual irrelevancies, and non-English papers. The challenge in collecting the articles for review was the status of the article, that is, whether it is open access or included in our institutions’ subscription or not.

**Figure 1:**
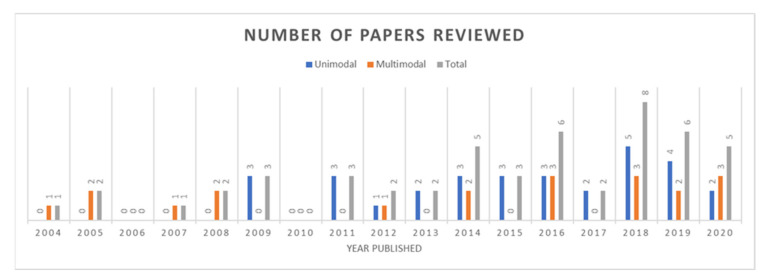
Overview of the years selected studies were published.

In total, for ECG-based emotion recognition, 51 papers were reviewed, and the distribution according to the year the papers were published is shown in Figure 1. The trend shows that the number of works increases by year, and this reflects the growing interest of researchers in this field. The overview also shows the number of ECG-based emotion recognition studies conducted with unimodal and multimodal approaches.

## 3. Theoretical Background

The contents covered here were cited from textbooks, academic journals, conference papers, and other sources with contextual benefits.

### 3.1. Autonomic Innervation of The Heart

**Figure 2:**
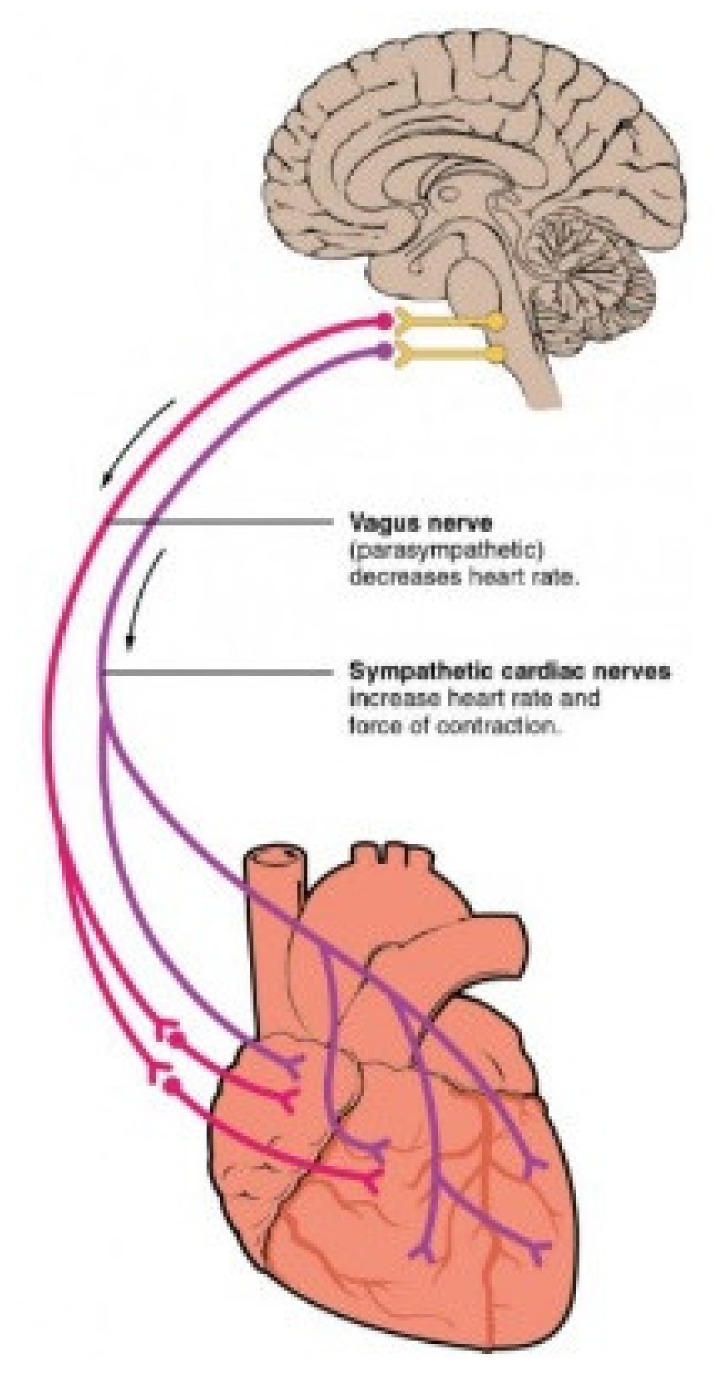
The ANS connection between the brain and heart [16].

The centers of the ANS’s control over the heart rhythm are located at the medulla oblongata [14]. Without any external factor, both centers provide an infinitesimal amount of stimulation to the cardiac muscle and cause it to have an autonomic tune. However, upon excitation, the cardioaccelerator releases the neurotransmitter norepinephrine and causes the HR to increase drastically. This process occurs throughout the SNS, as well as at the sinoatrial (SA) node, and is commonly known as the “fight or flight” response [15]. As for the decrease in the HR, the cardioinhibitory centers release the neurotransmitter acetylcholine (Ach) to the PNS. Metaphorically, this activation can be referred to as the “rest and digest” operation [15]. SNS and PNS stimulation flows through the cardiac plexus, cervical ganglia, and superior thoracic ganglia to the SA and atrioventricular (AV) nodes, with the nerves’ fibers reaching the atria and ventricles. Figure 2 shows the connection of the vagus nerve (PNS) and sympathetic cardiac nerves (SNS) in a simple model.

The physiological interrelation between the heart and brain communication influences certain characteristic changes when it comes to emotion. The ANS’s influence on emotional changes regulates various other body parameters [17]. According to the HeartMath Institute, the dynamic, continuous, and bidirectional communication of both organs affects one’s perception, emotion, intuition, and general health [13]. Hence, detecting the cardiac rhythm for emotion recognition purposes based on autonomic innervation is necessary in healthcare as a preventive measure towards negative emotions such as stress [18].

### 3.2. Electrocardiogram (ECG)

**Figure 3:**
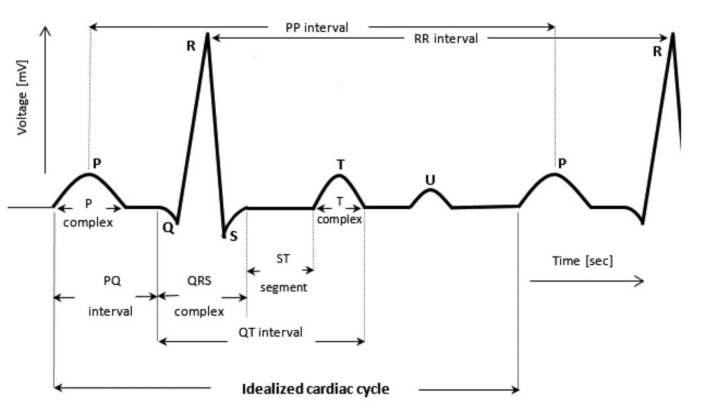
ECG cycle in a healthy and normal heart [28].

An ECG measures the electrical activity of the heart in different phases and perspectives based on the situation and configuration [19]. The signal acquired provides a graphical depiction of the deflection and wave series produced by each cardiac cycle, as shown in Figure 3. The main purpose of an ECG in clinics is to detect pathological cardiac conditions such as arrhythmia, heart disease, and epilepsy [20].

A normal ECG signal should have three segmented waves in a single cycle [19]. The first wave materializes from the atrial depolarization, and it is called the P wave. The second wave is the QRS complex, where it contains the highest amplitude caused by ventricular depolarization. The interval distance between R peaks is where the inter-beat interval (IBI) is usually calculated for HR detection [21]. Additionally, to extract HRV features from ECG signals, QRS detection is essential to sort out the RR intervals [22]. After a few milliseconds of plateau, a T wave appears because of ventricular repolarization [23], and the cycle repeats.

**Figure 4:**
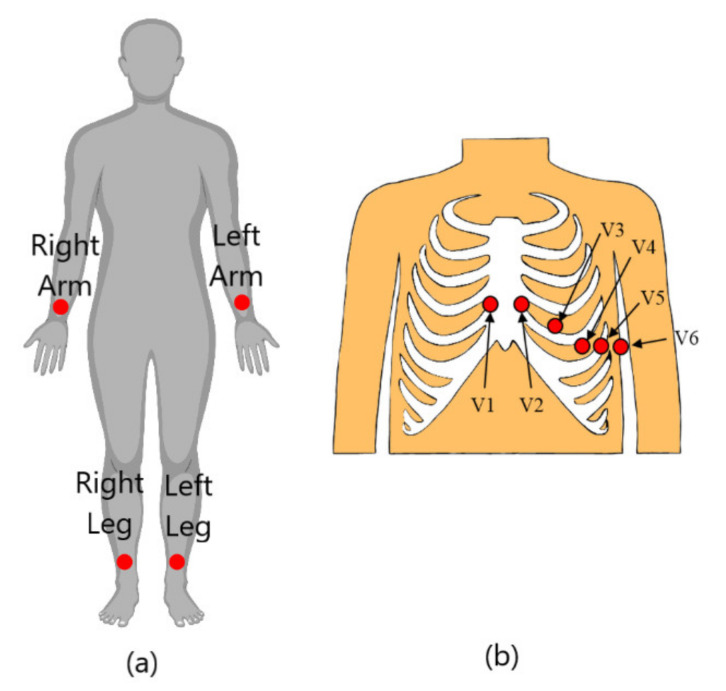
Possible electrode placements for ECG recordings [19]: (**a**) electrode placement for limbs lead configuration; (**b**) electrode placement for chest lead configuration [29].

According to Rattanyu [24], and Bexton et al. [25], ECGs are one of the most widely used biosensors in emotion recognition because of their quality, and the information on human emotions contained in the signals. Various studies have used ECGs as a single modality for emotion recognition. Theekshana et al. [26] stated that there are four prime reasons that ECGs alone are sufficient for an emotion recognition system. Firstly, ECG signals capture the heart activity, and ANS stimulation towards each emotion causes rhythmic changes in the heart [25]. Secondly, an ECG can be extracted using a less intrusive, mobile, and wearable device [27]. Thirdly, an ECG is a versatile biosensor that can collect data from different parts of the body: the chest or the limbs, as shown in Figure 4. Lastly, ECG signals have a higher amplitude among other biosignals [24].

### 3.3. Emotion Models

Emotion is a subjective and conscious mental experience accompanied by particular biological responses or changes [30]. Experts from different backgrounds have tried to uncover the universal definition of emotion; however, none of them have come to an agreement in establishing a single emotional model [15]. Despite this, the two most widely accepted and used emotional models are discrete categories and the affective dimension [1]. In addition, this paper also discusses another commonly used emotional model, the binary emotional model.

#### 3.3.1. Discrete Emotional Model (DEM)

The DEM categorizes emotions into standard terms such as joy, fear, anger, disgust, sad, funny, and neutral [31]. This emotional model is standardized and shared across languages and cultures [32]. Cicero and Graver [33] named 4 basic categories, while Ekman [34] summarized 6, and Izard [35,36] suggested 10 basic emotions. Although the number of emotion classes in the DEM varies, there are similarities between them. Among the emotion labels, the most common are happiness, sadness, and anger [20,37,38,39,40,41]. The reason for the three of them being selected the most is because of the prominent arousal level that can be easily detected compared to more relaxed emotions [22].

#### 3.3.2. Affective Dimensional Model (ADM)

**Figure 5:**
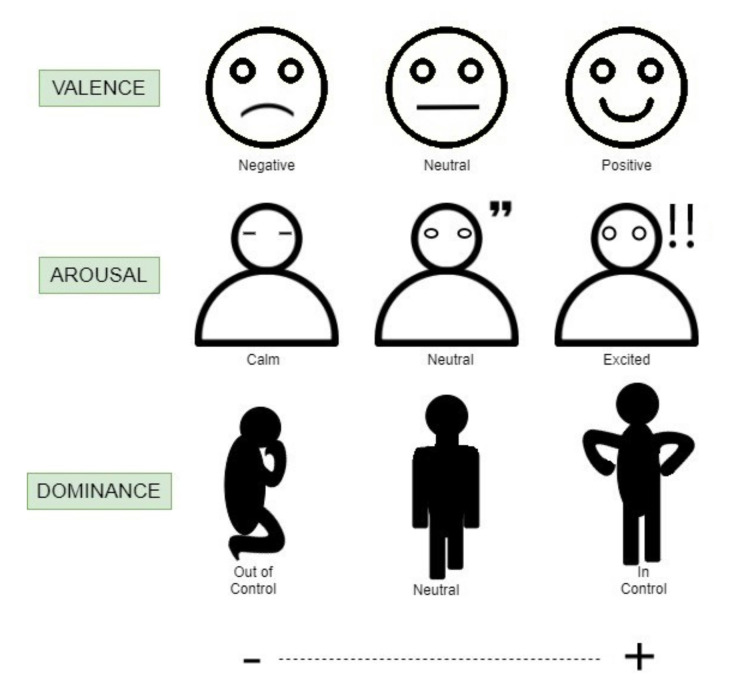
The graphical scheme provided to subjects to understand the ADM scales [45].

The ADM, which is also known as the continuous dimension model, is a range of two-dimensional planes of valence and arousal. One researcher preferred to add another plane of dominance into the model [42]. The ADM was developed by Russell [43] and has been adopted widely by researchers from different backgrounds. Figure 5 shows the illustration of valence, arousal, and dominance on a positive and negative scale. Valence is the feeling of pleasantness, either being appetitive or aversive, while arousal is the intensity of the feeling being experienced [44]. The dominance scale represents the authority to be in control, ranging from submissive to feeling empowered.

**Figure 6:**
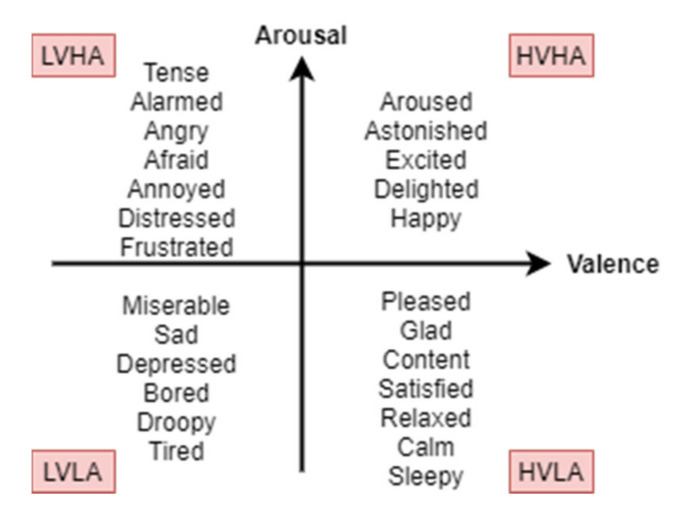
The mapping function between the ADM and DEM [46].

The versatility of the ADM compared to the DEM is demonstrated in Figure 6. Based on the valence and arousal scale, the categories of emotions can be segmented depending on the degree of intensity. High valence–high arousal (HVHA) is mapped to excitation, while high valence–low arousal (HVLA) is mapped to feeling calm, or relaxation. Low valence–high arousal (LVHA) is considered as anger and feeling distressed, while low valence–low arousal (LVLA) is related to sadness and feeling depressed. The middle of the scale is considered as a neutral state.

#### 3.3.3. Binary Emotional Model

**Figure 7:**
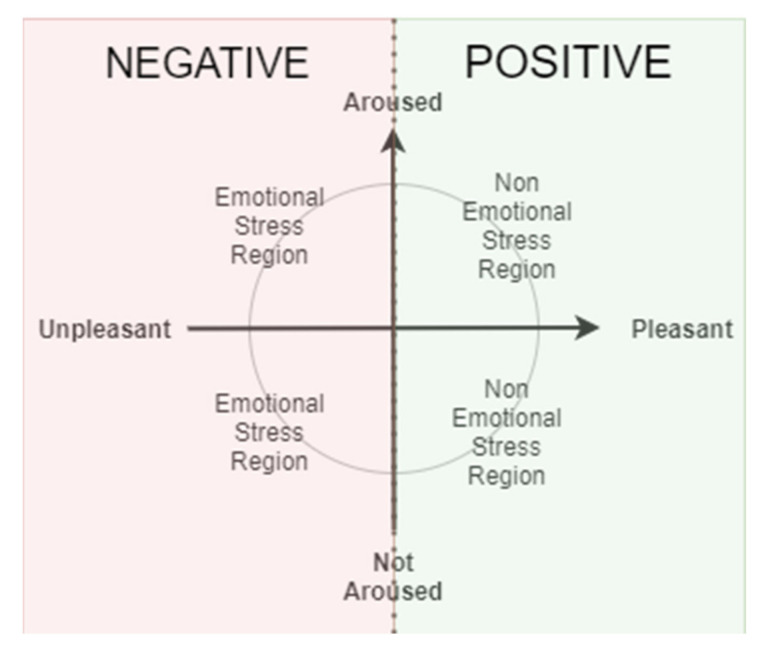
Pos/Neg as a model that identifies between good (no stress) and bad (stress) emotions.

The binary emotional model consists of positive and negative emotional states (Pos/Neg) [47]. The purpose of this model is to simply generalize between which emotions are bad and which emotions are good. Negative emotions may cause mental stress to the bearer and the people around them. It is unhealthy to be exposed to prolonged negative emotions as it affects the physiological state of a person. Depression, anxiety, and bipolar disorder are known effects of emotional and mental stress [48,49]. Moreover, by simplifying the emotional model to two classes, a targeted application of an emotion recognition system can be built with less complexity. A higher accuracy of training and testing models can also be expected. Figure 7 shows the emotional stress model proposed by [39]. Instead of valence, the author used a pleasantness scale to describe the region of potential mental stressors. Any emotions categorized under negative valence such as sadness, anger, fear, and disgust are potential stress factors that may lead to complications. Thus, the binary emotional model is another important classification model for affective computing studies.

### 3.4. Emotion Elicitation

Inducing basic emotions for data collection in an experiment requires certain guidelines and standard operating procedures. There are five common elicitation techniques which are audio visual, imagery, music, memory recall, and the situational procedure [50]. The less common approaches are naturalistic conversations or debates [51], driving [52], video games [53], and virtual reality [54].

Audio visual techniques can be segmented film clips for targeted emotions, or videos with the same purpose [31,45,55,56,57,58,59]. The length of the videos varies, as does the length of the recorded physiological signals. Imagery is the act of reading vignettes [50] and experiencing deep emotions through contemplation [60], but in addition to that, pictorial images such as the International Affective Picture System (IAPS) [61] have been used widely too. Music listening is another popular way to activate emotions through the lyrics, melody, and tempo variations [62]. The renowned dataset for affective audio stimulation is the International Affective Digitized Sounds system (IADS) [63]. Memory recall involves remembrance of personal experiences to reactivate the essence of emotions circa that moment [64]. The situational procedure necessitates fabricating a social environment that elicits the targeted emotion.

As it was described in [50], the most effective way to induce basic emotions is through audio visuals. Imagery is effective for happiness, surprise, fear, and anger. Music is only effective for happiness, sadness, and fear. Memory recall is recommended to induce happiness, anger, disgust, sadness, and fear, but not surprise. Finally, the situational procedure is a good approach for happiness, anger, fear, and surprise.

### 3.5. Emotion Evaluation

Emotion evaluation is an annotation perspective for emotion labeling on the data collected. The most common approach is through a first-person perspective or self-assessment. In this way, the subject personally labels their emotions on a Self-Assessment Manikin (SAM) [65]. The questionnaire varies depending on which emotional models are used. Usually, there will be a pictorial description of emotions and the intensity scale to ease the labeling process, as shown in Figure 5. The problem with internal annotation is that the subject might feel discomfort and insecure in sharing their true conscious and unconscious experiences towards the stimuli [15]. This indirectly reduces the reliability of the reported emotional experience.

Another perspective for emotion annotation is implicit assessment or external evaluation. This can be conducted through a second-person perspective and third-person perspective. The second-person perspective is someone who watches the subject experience the stimuli in real time and labels what they think the subject feels [51]. Meanwhile, third-person perspectives are external, conducted by watching the recordings of the subject’s facial expression and body gestures, and then only annotating the guesses on what emotions the subject feels. Both methods have a disadvantage of bias, and they can easily be deceived [15]. Their perception often depends on personality, cultural bias, and environmental attributes.

## 4. ECG-Inclusive Affective Datasets

Affective datasets that have been collected using various physiological modalities are available in academic archives. Although they are not standardized, there are still commonalities between them. Since this review paper is only interested in ECG-based emotion recognition systems, the datasets enlisted are ECG-inclusive modalities. The focus is on the summary of the stimulation used, the data size, the modalities included, the ECG device used, the ECG configuration, emotional annotations, the model, and perspectives. Among the datasets with ECG signals are the following:

1. **AMIGOS** [55]: This stands for **A** dataset for **M**ultimodal research of affect, personality traits, and mood in **I**ndividuals and **G**r**O**up**S**. The data were collected from 40 subjects watching videos, with 16 samples each. Biosignals included are ECG, EEG, and GSR. The ECG device used was a Shimmer, at a 256 Hz sampling frequency. The ECG lead configurations used were right arm left leg (RA-LL), and left arm left leg (LA-LL). The emotion annotation labels were from a self-assessment, and third-person perspectives with a 3D ADM.
2. **ASCERTAIN** [56]: This stands for a multimodal datab**AS**e for impli**C**it p**ER**rsonali**T**y and **A**ffect recognit**I**o**N** using commercial physiological sensors. The data were collected from 58 subjects watching 36 video clips. The physiological signals used were ECG, EEG, and GSR. For ECG, the sampling rate was 256 Hz, with two unspecified lead configurations. The emotion annotation perspective was only from self-assessment, and the model used was the ADM on a scale of valence and arousal.
3. **AuBT** [66]: This stands for **Au**gsburg **B**iosignal **T**oolbox by the University of Augsburg. It contains a MATLAB GUI for emotion recognition purposes, together with a data corpus recorded from ECG, EMG, skin conductance (SC), and respiration (RSP). The data were from a single subject, with 100 samples collected within the span of 25 days while listening to music of the subject’s choice. The ECG signal sampling rate was 256 Hz, with only one lead configuration. The emotions were labeled by self-assessment using the DEM. The four classes of emotions are joy, anger, sadness, and pleasure.
4. **CASE** [67]: This stands for the **C**ontinuously **A**nnotated **S**ignals of **E**motion. The data were collected from 30 subjects in real time while watching various videos. The physiological modalities included are ECG, BVP, EMG, and GSR (EDA). The ECG device used was from Thought Technology, and the configuration setup had three leads, 1 kHz. The annotation was by self-assessment using the ADM.
5. **CLAS** [68]: This stands for **C**ognitive **L**oad, **A**ffect and **S**tress Recognition. The data were collected from 62 subjects, with 32 samples each. The stimuli were separated equally between video clips and IAPS pictures. The biosignals included are ECG, PPG, and EDA. The ECG device used was the one-lead Shimmer3, with a right arm left arm configuration. The sampling rate was 256 Hz. Self-annotation of the valence and arousal ADM was performed by the subjects.
6. **DECAF** [57]: This stands for a multimodal dataset for **dec**oding user physiological responses to **af**fective multimedia content. The data were collected from 30 subjects with 76 samples. Here, 40 of the 76 samples were from music videos at a 1 min cap, while the others were from watching movie clips. The biosignals included are ECG, EMG, magnetoencephalogram (MEG), and electrooculogram (EOG). The sampling rate for the ECG was 1 kHz, and it was downsampled to 256 Hz. A one-lead configuration was used for this setup. The annotation was from a first-person perspective, and the ADM with a 3D scale was implemented.
7. **DREAMER** [58]: This dataset contains data collected from 23 participants, with 18 samples each. The stimuli used were video clips ranging from 1 to 3 min, with the focus on the ECG and EEG modalities. The ECG device used was a low-cost, wireless, portable, and wearable off-the-shelf device from Shimmer. The sampling rate was 256 Hz, with two-lead and three-lead configurations. Self-annotation of the subjects was conducted using a valence, arousal, and dominance ADM.
8. **DSDRWDT** [52]: This stands for **D**etecting **S**tress **D**uring **R**eal-**W**orld **D**riving **T**asks. The data were collected from 24 subjects while they were driving in a real-world condition. The biosignals included are ECG, EMG, SC, and RSP. The ECG device used was a FlexComp, with a 496 Hz sampling rate. The lead used was right arm left leg (RA-LL). The drivers labeled their stress levels through three stages: low, medium, and high. The emotional model considered was the Pos/Neg category model.
9. **EMDC** [69]: This **e**motion-specific **m**ultilevel **d**ichotomous **c**lassification dataset contains signals collected from 3 subjects, with 360 samples of music listening. The physiological modalities included are ECG, EMG, SC, and RSP. The ECG device used was a three-lead Procomp^2^ Infiniti, at a 256 Hz sampling frequency. The affective annotations were from self-perspective with a 2D ADM.
10. **K-EmoCon** [51]: This dataset contains data collected from 32 subjects in real time from a naturalistic conversation (paired debates on social issues) to induce emotions. The physiological modalities included are ECG, EEG, BVP, EDA, and skin temperature (SKT). For the ECG signal, a Polar H7 was used, at a 1 Hz sampling rate. The only feature extracted was the HR. This paper claims to be the first publicly available dataset on emotion recognition that has a multi-perspective annotation from self-assessment, second person and third person. The ADM with valence and arousal scales was implemented.
11. **MANHOB-HCI** [59]: Data were collected from 27 subjects, with 20 samples, using ECG, EEG, GSR, EDA, RSP, and SKT. The ECG device used was a Biosemi Active II, with a three-lead configuration. The sampling rate was 1024 Hz and was downsampled to 256 Hz. Based on the emotional videos watched, the subjects self-reported their affective state with a 3D ADM.
12. **MPED** [31]: This stands for **M**ulti-Modal **P**hysiological **E**motion **D**atabase. The data were collected from 23 subjects, with 28 samples, watching video clips less than 5 min each. The biosgnals included are ECG, EEG, GSR, and RSP. The Biopac System with three-lead configurations and a 250 Hz sampling frequency was used for the ECG signal acquisition. The annotation perspective was from the first-person view using seven classes of the DEM: joy, funny, anger, fear, disgust, sad, and neutral.
13. **SWELL** [70]**:** This dataset is also known as SWELL knowledge work (SWELL-KW), and it is a new multimodal dataset for research on stress and user modeling. The data were collected from 25 subjects performing tasks such as writing, presenting, reading, and searching to elicit stress. The physiological signals recorded were ECG and SC. The ECG was recorded through a Mobi device (TMSi), with the electrodes placed in a triangular configuration on the chest. The sampling rate was 2048 Hz, with three leads attached. The assessment was conducted by the subjects through labeling two emotional models, which were the ADM and Pos/Neg.
14. **WESAD** [71]: This stands for **We**arable **S**tress and **A**ffect **D**etection. The data were collected from 15 subjects watching video clips and provided with public speaking and mental arithmetic tasks. The biosignals included are ECG, BVP, EDA, EMG, RSP, and temperature (TEMP). The ECG signal was acquired from a RespiBAN Professional using a three-lead configuration. The sampling rate was 700 Hz. The subject self-annotated their emotions using a three-class Pos/Neg model. Amusement, neutral, and stress were the classification categories implemented.

All of these ECG-inclusive datasets are summarized in Table 2. The stimulus used to induce the emotions during data collection, the data size, available modalities, details of the settings of ECG collection, the emotion annotations, the model, and perspectives are tabulated.

### Dataset Popularity Analysis

**Figure 8:**
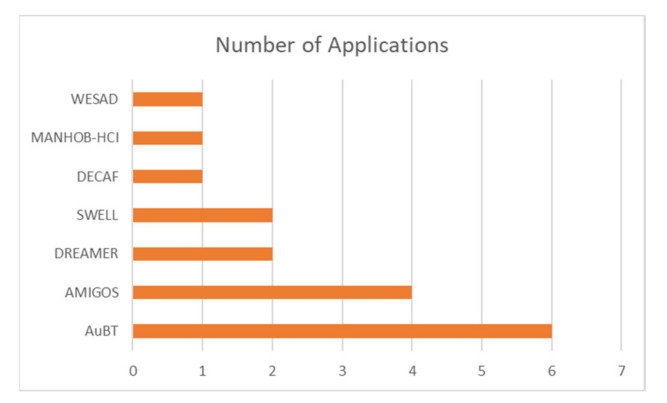
The number of times datasets were applied in different research studies found in the summarized literature.

Even though multiple datasets have been proposed and made available for others to use, not all datasets have been adopted by other researchers. Hence, based on the summarized literature from this review, the number of times a dataset has been adopted and cited in other studies (excluding self-citation) was calculated and is plotted in Figure 8. The most popular dataset being used for emotion recognition studies using ECG, as observed here, is AuBT, with six adoptions. Although the database was published in 2005, the citations observed here came from 2016 onwards. The popularity of the AuBT dataset is followed by AMIGOS, with four adoptions from 2018 to 2020. Third place goes to DREAMER, with two adoptions in 2020 and 2019. SWELL was published in 2014, but the adoption of the dataset is only found in two papers from 2020. The other three mentions are DECAF, MANHOB-HCI, and WESAD. All three have one adoption and citation in other research studies. Other datasets such as ASCERTAIN, CASE, and CLASS are not found in any other studies by far. Many of the works reviewed used their own collected data.

## 5. Development of Emotion Recognition Systems

**Figure 9:**
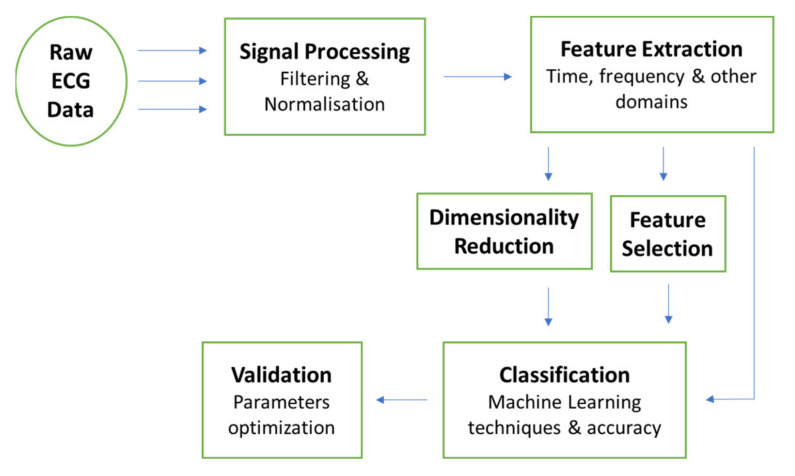
General methods for an ECG-based emotion recognition system using machine learning.

There are several steps in developing emotion recognition systems. This work focuses on the development of emotion recognition systems using machine learning techniques. The first step is pre-processing, which is to clean the signal from unwanted noises. Next is feature extraction using various techniques. The usage of feature selection as well as feature reduction to find the relevant emotion-related features is optional and can be included after feature extraction. The last step is classification and validation techniques using machine learning algorithms. The common adopted pipeline of emotion recognition models is presented in Figure 9.

### 5.1. Pre-Processing

An ECG signal is considered as a high-sensitivity physiological signal with a low recording voltage between 0.5 and 5 mV [72]. Generally, the signal is susceptible to noise and corruption due to various internal and external factors depending on the method of application. The main sources of ECG noise are power line interference, muscle movements, electrode–skin contact, motion artifacts, baseline wander, electronic and electromagnetic device interference, external electrical system interference, internal high-frequency noise, and respiration or bowel sounds. The common frequency for muscle movements is 5–50 Hz, 0.12–0.5 Hz (at 8–30 beats per minute) for respiratory, 50/60 Hz on AC electrical systems, and >10 Hz on other electrical and electronic devices [73]. Although there is a wide variety of ECG filters, the applications depend on specific needs to denoise and reduce the amount of information complexity towards a desired level.

The multiple-configuration Butterworth filter is the most widely used filter based on the summarized papers. In [47,74], a low-pass Butterworth filter with a 60 Hz cut-off frequency was applied to remove a higher background noise of ECG signals. A 0.05–100 Hz Butterworth bandpass filter was used in [69] to remove noise, while a 49–51 Hz band-stop Butterworth filter was used in [75] for power line interference at 50 Hz. According to [72], although the bandpass filter may remove most of the stated noises, solely depending on it is discouraged as the result might not be the best. A fourth-order Butterworth filter with a 100 Hz cut-off frequency [76] and a sixth-order Butterworth filter with a 45 Hz cut-off frequency [40] were used to remove high-frequency noise and powerline interference. The lowest order of the Butterworth filter works best in the time domain, while in the frequency domain, a higher order is better.

In removing a high-frequency interference, [75] applied a 1–60 Hz bandpass filter, while [77] used a 5–15 Hz bandpass filter. In [24], an interpolation filter was utilized to remove signals of 30 Hz and below. A notch filter or a band-stop filter was applied in [20,78] at 50 Hz. A second-order infinite impulse response (IIR) notch filter was used to eliminate powerline noise and motion artifacts in [47]. A fourth-order notch filter at 50 Hz was used in [76] to eliminate power line interference, as suggested by [79].

The most common frequencies in ECG signals that should be preserved for further processing and feature extraction are 0.67–5 Hz (at 40–300 bpm) for detecting the HR and P wave. The QRS complex can be detected within 10 to 50 Hz, and the T wave at 1–7 Hz. A high-frequency potential may also be considered at 100–500 Hz [73]. To determine which filter is best to be used, the frequency setting and calibration pulse should always be informed first so that the ECG signal can be interpreted accurately.

### 5.2. Feature Extraction

ECG feature extraction has different approaches depending on the way raw signal calculations can be manipulated into meaningful information. This section begins with the most basic ECG signal processing through PQRST detection and the extraction of statistical features. Next, feature extraction for the HR and within beat (WIB) features is explained. The third part summarizes HRV and IBI as the most used features from ECG modalities to detect human emotions through ANS activity within the heart. The last part summarizes other feature extraction techniques used throughout the literature reviewed.

#### 5.2.1. PQRST Detection and Statistical Features

The most basic features to be extracted from ECG signals are the PQRST points’ allocations. Between the P wave, QRS complex and T wave, the QRS complex was considered important in defining the HR and HRV through IBI calculation [55,80]. The Pan–Tompkins QRS detection algorithm [81] is considered as the most common technique to find the R peak location [58,67,69]. In [39,40], the QRS complex was derived by applying a nonlinear transformation on the first derivative (Gaussian first-order differentiator) of the filtered ECG signal [82,83]. Continuous wavelet transforms (CWT) are applied to detect a precise R location and then the QS, P, and T waves [84]. Finally, in [83], a built-in R peak detection was embedded in Acknowkedge3.8.2 application software, and there is no need for the researcher to manually extract the features.

Based on PQRST detection, individual statistical features can also be extracted [58,66,85,86]. The statistical features extracted include mean, median (med), standard deviation (std) and quartile deviation, minimum (min), maximum (max), and range (max-min) of individual P, Q, R, S, and T. The authors of [84] extracted only the amplitude of P, R, and S, before proceeding to analyzing the other features.

#### 5.2.2. HR and WIB Features

HR is measured in beats per minute (bpm). Considering that one cycle or one beat can be measured between two successive R peaks, the HR can be derived simply through averaging the overall signals collected through a period. The HR is proven to show distinct feature changes [87] and has been used in various ECG-based affective studies [6,24,39,51,53,55,56,57,67,71,75,78,85,88]. The benefits of HR over other features are the simplicity of the calculation and not requiring a highly accurate measurement. Even during an intensive exercise, the measurement of the HR is still reliable.

WIB features were proposed by [24], which calculate the statistical values of ECG intervals. Mean, med, max, min, and standard deviation are calculated from PR, ST, and QRS intervals [26]. Instead, in [58,66,85,86], PQ, QS, and ST intervals were used to calculate the statistical features stated, with an addition to the range. QRS morphologies were extracted in [89] based on clinical application. The morphology features are *qrsWBR* (width between R peaks and the next Q), *qrsWRE* (width between S and R peaks), *qrsABR* (difference between amplitude of R peaks and the next Q), *qrsARE* (difference between amplitude of R peaks and the consequential S), and *qrsMOR* (the shape of the QRS interval).

#### 5.2.3. HRV and IBI Features

HRV measures specific changes between heart beats in the time domain. The time between beats is measured in milliseconds (ms) and is called an RR interval or IBI. The variation in IBI values contributes to the readings of HRV. HRV features are claimed to be one of the most used methods in ECG-based emotion recognition systems [69,90]. HRV is also known to have distinct changes in emotion variations [87] and used as an indication of stress and mental effort in healthy adults [69]. Moreover, HRV is the most precise non-invasive physiological technique in measuring the activity of the ANS throughout the body. The widely available and affordable consumer-grade ECG devices that can record a significantly good signal are sufficient for HRV feature extraction.

**Figure 10:**
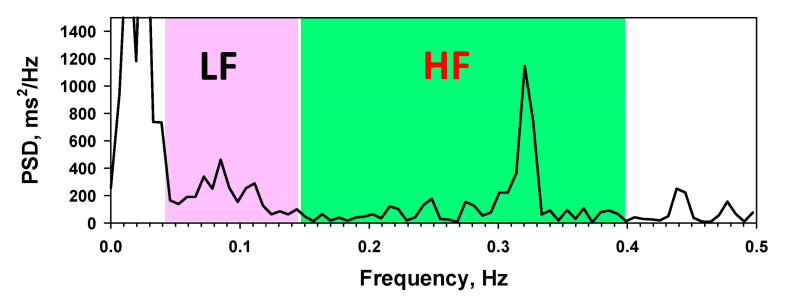
Power spectral density (PSD) features [97].

Out of the 51 studies summarized, 31 of them used HRV, with a slight common variation. However, in general, there are three domains of HRV feature analysis: time domain, frequency domain, and time–frequency domain. A detailed explanation of each domain is presented below:

- **Time domain** [26,91,92] (**Temporal** [15]): This measures the amount of variability in IBI, where the expression comes in the form of a natural logarithm (Ln) of original units, or the original units themselves, for a more normally distributed formation. There are short-term indices for recordings around minutes in length, and long-term indices which usually record over a period of 24 h. The first feature matrix is the standard deviation of the normal-to-normal interval (SDNN). This feature is represented in the unit of milliseconds (ms) for a standard short-term recording of 5 min [93], and 60 to 240 s for ultra-short term recordings [94,95]. SDNN changes also correlate with SNS and PNS activity in the heart. Next, the standard deviation of RR peaks (SDRR) is very similar to the previous case, but it includes false and abnormal beats measured at R peaks. NN50 and pNN50 are the number of adjacent normal-to-normal intervals and percentage of them that are more than 50 ms. These features are known to accommodate PNS activity in the heart [96]. Other variations are NN20 and pNN20, respectively. Next, the root mean square of successive differences (RMSSD) is an index of IBI variance in the HR. Finally, the HRV Triangular Index (TriInd) feature is usually combined with RMSSD to detect pathological cardiac complications, and triangular interpolation of a normal-to-normal interval histogram (TINN) is used as a histogram baseline for a normal-to-normal interval.
- **Frequency domain** [26,91,92] (**Spectral** [15]): This measures the amount of power at various frequencies using fast Fourier transformation (FFT). The amplitude of FFT can then be derived into a power spectral density (PSD). In spectrogram analysis, there is a range of feature levels available such as ultra-low frequency (ULF), very-low frequency (VLF), low frequency (LF) and high frequency (HF), as shown in Figure 10. However, in the emotion recognition system, ULF and VLF are not utilized as both need at least 24 h of ECG recording, which is not practical for emotion recognition. VLF, LF, and HF bands have a window range from 0.0033 to 0.04 Hz, 0.04 to 0.15 Hz, and 0.15 to 0.40 Hz. All three correlate with SNS and PNS activity changes. In fact, a low HF power reflects negative emotions such as anxiety, worrying, stress, and panic. Based on the bands, there are also variations of the normalized LF and HF, the LF/HF ratio, and the total spectral power. Other statistical features that have been extracted from the frequency bands are spectral centroids, spread, kurtosis, skewness, slope, variation, decrease, roll-on/off, and total energy.
- **Nonlinear domain** [15,91] (**Geometrical** [15,26]): This measures the nonlinearity of time series of the unpredictability of the HRV complexity mechanism. The features are extracted from Poincare geometric plots and allow a refined pattern detection through a scatter plot. The parameters are the area of the total HRV eclipse (S), each point, the standard deviation from both axes (SD1), the standard deviation of each point from both axes plus the RR interval (SD2), and SD1/SD2. The feature variation includes SD12, Area0, Area1, Area2, Area3, and Area4.

#### 5.2.4. Empirical Mode Decomposition, Wavelet Transform, and Fourier Transform

Empirical mode decomposition (EMD), also known as the Hilbert–Huang transform (HHT), is a technique to transform signals into parts called intrinsic mode functions (IMF) [98]. This technique is suitable for nonlinear and nonstationary signals such as those from an ECG. With the IMF characteristic, the instantaneous frequency and amplitude of the signal can be defined. Moreover, the HHT also preserves the characteristic of frequency changes as the lengths of original signal and IMF are the same. The application of EMD for ECG feature extraction techniques to emotion recognition systems is seen in a few papers such as [21,26,54,76,99,100]. In [54], 35 features were extracted from IMF1 and IMF2. The features consist of statistical features such as mean, max, standard deviation, variance, skewness, kurtosis, and others.

The wavelet transform is a technique for multiresolution analysis [101] and divided into two forms. The continuous wavelet transform (CWT) has the capability of extracting features from the signal with the determination of extremum points and inflection points, while the discrete wavelet transform (DWT) can extract statistical and stochastic characteristics, and the energy spectrum. In general, the wavelet transform decomposes data into different frequency and time scales using a mathematical transformation function. The computing process involves dilation and translation of functions, or multiscale refinement of signals. The wavelet transform is also known to be able to solve difficult problems that Fourier transforms are not capable of [102]. In [84,101], the CWT is used to perform the feature extraction on ECG signals, while [89,103] applied the DWT in their framework process.

The Fourier transform is another technique for decomposing functions that are dependent on the time of space into functions that are dependent on the temporal or spatial frequency. The two common Fourier transforms in emotion recognition studies are the discrete Fourier transform (DFT) and the FFT. They are almost identical methods, with the FFT being a more efficient function, where the computation performs faster than the DFT. Again, in [76], the authors combined EMD and the DFT as IMF alone does not contain much information to provide any distinctive features. Another adoption of the DFT is also found in [26], where the application of feature extraction is paired with EMD and other methods. Finally, application of the FFT is only seen in one paper [69], where the features were derived from a partitioned coefficient within the frequency range into overlapping sub-bands with the same bandwidth. From that, the sub-band spectral entropy (SSE) is computed to identify the disorganization or uncertainty in a random variable. This helps the pattern recognition by scaling the intensity of a classifier’s confidence.

#### 5.2.5. Others

There are some independent feature extraction techniques based on ECG signals used for emotion recognition systems. Various novel approaches have been proposed to perform the task with the aim of extracting useful feature information that is relevant to the ANS activity of the heart. The prospective approach has been taken, from the mathematical process derivation function to pictorial plotting and statistical feature analysis.

Detrended fluctuation analysis (DFA) and detrended cross-correlation analysis (DCCA) were applied in [104]. Features from the multifractal spectra were also extracted in that paper. DFA is categorized under nonlinear feature analysis, and the work in [105] also applied this method along with Poincare plot feature extraction from HRV.

In [20], Coiflets wavelets (Coif5) at level 14, the discrete cosine transform (DCT), and Daubechies wavelet (db4) at level 8 were applied before using matching pursuit coefficients for feature extraction. The features extracted were statistical such as mean, variance, standard deviation, minimum, and maximum.

Instead of using the numerical values of ECG signals to extract the features, a graphical plot and image pattern recognition were applied in [47]. The methods used were the local binary pattern (LBP) and the local ternary pattern (LTP). The LBP is widely used in computer vision and image processing research, particularly in facial recognition. The LTP is the modification of the LBP by changing it from a binary operation of 1-0 to three operations of -1-0-1. The operation depends on the frame length and frame shift to extract the features.

Another method that has been reported is feature extraction through the Nonlinear Autoregressive Integrative (NARI) Point-Process Model [106]. The analysis of heartbeat dynamics started from detecting RR peaks, and following the Wiener–Voterra representation, a specific point process model was created for instantaneous identification up to the third order. The features are extracted from Lyapunov exponents as well as instantaneous spectra, and spectra. This evaluation is also known to be in the realm of high-order statistics (HOS).

A nonlinear approach based on Hurst was proposed in [40] by using rescaled statistics (RRS) and finite variance scaling (FVS). The new Hurst features are combined into HOS to be classified into six basic emotional states. The value of Hurst can also be obtained by EMD, the wavelet transform, and finite variance scaling. Before applying the feature extraction procedure, the QRS complex is extracted for further computation of RRS and FVS. In this process, six features are extracted from each sample in the study.

Other ECG feature extraction methods found in the reviewed works are the multi-variant correlation method and spectrograms. In [107], the authors applied a linear multi-variate approach for their feature function analysis. Meanwhile, in [108], the author extracted the features using deep learning by converting time series data to frequency domain-based images. Based on the images, only the 0–5 Hz range was converted into a spectrogram, and the data were fed into a VGG-16 network. Finally, 4096 features were extracted and studied.

### 5.3. Feature Selection and Dimensionality Reduction

Extracted features do not promise fully relevant correlations with physiological changes in emotion regulation. Feature selection is a method to optimize the classification architecture by only picking the best feature combinations and eliminating noninformative features. This can also reduce the computational cost of the classification in the later step. In [26], recursive feature elimination, the chi-square test, the P test, random forest feature selection (RF FS), extra tree feature selection, and random support vector machine feature selection were used. Moreover, swarm intelligence is also common in the feature selection process. The author of [74] applied the genetic algorithm, while ant colony optimization was used in [104]. Binary particle swarm optimization (BPSO) and hybrid particle swarm optimization (HPSO) have also been applied for feature selection [84]. The wrapper method and the Tabu search algorithm are found in [77] and [103]. In [109], the author used Kullback–Leibler divergence as a feature selection. Other common techniques are sequential forward selection (SFS) and sequential backward selection (SBS), which have been applied in [86,87,110].

Dimensionality reduction is a technique to reduce the number of features by transforming a higher dimension feature matrix into a lower dimension without losing the necessary information. The two most used techniques were principal component analysis (PCA) and linear discriminant analysis (LDA). The transformation of PCA is unsupervised, while LDA is supervised. The applications of PCA were viewed in [20,55,67,85,89,108,111]. LDA, also known as Fisher’s linear discriminant analysis, was used in [20,24,53,87] as a dimension reduction procedure.

The applications of feature selection and dimensionality reduction techniques stated are reported to be beneficial in terms of improving the training and testing accuracy for emotion recognition systems. Moreover, the time taken to perform the classification is reduced significantly as less data need to be processed at a time. Finally, the chance to overfit the trained model is reduced, as the noisy data are eliminated from the final data fed to the classifier.

### 5.4. Classification

Classification techniques are divided into two main categories which are machine learning and deep learning. Commonly, if deep learning is adopted in physiological-based emotion recognition, there are no feature extraction and feature selection steps. If the deep learning architecture has a convolutional layer, it might somehow be considered as a dimensionality reduction stage.

Machine learning methods are divided into three learning categories which are supervised learning, unsupervised learning, and hybrid learning. In affective computing, the majority of the research adopted supervised learning through emotion labels such as ADM, DEM, and Pos/Neg through SAM. However, there is one work that used unsupervised learning, which is [112]. The ECG signals were unlabeled, and the convolutional neural networks (CNN) were trained to find any signal transformation for emotional patterns. Then, the weights were passed on to the labeled data for testing. The accuracy shows a significantly better result than most of the supervised learning techniques.

A classifier that has been frequently adopted and performed the best in emotion recognition systems is the support vector machine (SVM) [15]. From 24 out of the 51 studies summarized here (presented in the following section), SVM was adopted as either the only classifier or one of the machine learning algorithms to be compared. SVM kernels are simply the methods or behavior of making the hyperplane decision boundaries work in certain manners. In [89], SVM constantly performed better than random forest through every ratio of generated emotional data in the training set.

Although SVM is popular, it is not always the best classifier, as reported in several works. Other well-performing classifiers used are k-nearest neighbour (KNN) and naïve Bayes (NB). KNN was reported to perform better than SVM in [39,77]. Meanwhile, [56] showed that NB performed better than SVM in both valence and arousal classification using a single ECG modality. Classifiers that were also reviewed are decision tree (DT), random forest (RF), AdaBoost (AB), gradient boost (GB), quadratic classifier (QDA), and LDA. For less known classifiers such as extra tree, regression tree, and ensemble bag tree, their performance was reported to be considerably good in [26] when compared to RF and GB.

Neural network-based deep learning classifiers come in different forms and configurations. Based on the literature, there are a lot of neural network (NN) infrastructures such as 1-NN, deep convolution neural network (DCNN), probabilistic neural network (PNN), backpropagation neural network (BPNN), radial basis function neural network (RBFNN), multilayer perceptron (MLP), and extreme learning machine. Extreme learning machines alone were shown to improve the training accuracy of many databases [108]. DCNN also showed classification accuracy of the AMIGOS dataset in [113] for valence and arousal. The best accuracy was shown in [20] using PNN to classify five-class and three-class DEMs. However, the study was subjected to a credibility request as the result might be biased by overfitting.

### 5.5. Validation

Validation is a crucial step in building a machine learning model, especially when dealing with a subjective application such as emotion recognition. This step is designed to see the overall performance of the trained models when it comes to new data. The partitioning between training and testing datasets is to ensure the model can perform a validation step by imitating real-world scenarios outside of the experiment setup [15]. The generalization ability of validation allows the model to increase variability and reduce overfitting. The most common validation techniques are called cross-validation (CV) with different versions of approaches.

Non-exhaustive cross-validation of k-CV is a resampling procedure conducted with k number of folds to reshuffle and train the limited data sample, with 5 and 10 being the standard number of k when it comes to the number of folds in k-CV. When k is bigger than that, the subjected models are considered biased. The 5-fold CV was practiced in [54,74], while a rare 15-fold CV was only conducted in [54]. Moreover, 10-fold CV is the most widely practiced cross-validation technique, with 12 papers in total [6,26,39,47,53,54,55,88,99,112,114,115].

Exhaustive cross-validation techniques have two main variations. The first is leave-one-out cross-validation (LOOCV), where the models are tested and validated from end to end without leaving one participant or subject as a final validation. This method takes more time than leave-one-subject/participant-out cross-validation (LOSOCV/LpO CV). The main advantage of exhaustive CV over non-exhaustive CV is the lower bias as it trains the possible validation combination across all datasets. However, considering a large amount of computational work, the validation process takes a significantly longer time to complete. LOOCV was applied in [55,56,68,69,77,106,109,116], while LOSOCV was adopted in [71,105,110].

## 6. Review of ECG-Based Emotion Recognition Systems

The 51 reviewed works are summarized in Table 3 and Table 4. Table 3 summarizes 31 studies on combinations of unimodal and multimodal ECG-based affective research that reported on ECG standalone results. Meanwhile, Table 4 summarizes 20 affective research studies that included ECG as one of their physiological modalities but did not mention the classification accuracy of using solely ECG as the input. In this section, the works that achieved more than 90% accuracy are highlighted.

In Table 3, there are seven works that reported more than 90% accuracy in classifying emotions based on varying emotional models. Firstly, Sarkar and Etemad [112] performed a self-supervised emotion recognition study using four datasets which are AMIGOS, DREAMER, WESAD, and SWELL. Based on the raw ECG signals from each dataset, the neural network learned high-level abstract representations, and the weight was transferred to an emotion recognition network. The results show an improved performance compared to fully supervised learning. Although AMIGOS and DREAMER did not manage to pass 90% and above accuracy, WESAD and SWELL were claimed to be successfully classified, with accuracy above 90%. With 96.9% accuracy, the author managed to classify WESAD with the Pos/Neg Model. Moreover, with 97.3%, 96.7%, and 93.3%, the author managed to classify SWELL on a model based on a binary scale of valence, arousal, and stress.

In a study conducted by Zhang et al. [104], the data were labeled according to a DEM with four classes of emotions of happy, sad, pleasant, and angry. The overall accuracy based on the ECG unimodal approach was reported to be 92%. The individual accuracies were 97%, 92%, 91%, and 88% for angry, sad, happy, and pleasant. The best classification results among three classifiers were achieved using KNN from two sets of extracted features. The first feature set consisted of the time and frequency domains, with statistical characteristics of ECG signals, while the second set of features was correlation features. The correlation features were inclusive of the autocorrelation feature parameter, cross-correlation feature, and multifractal feature parameters. The feature selection used was the max–min ant system, which is a derivation of ant colony optimization.

Goshvarpour et al. [20] conducted an emotion recognition study based on ECG and GSR collected from 11 subjects that listen to music as an affective stimulation method. The result analysis was taken from the perspective of performance comparison between ECG and GSR unimodal approaches. Based on the matching pursuit method, three dictionaries were applied for feature extraction on the raw ECG signals, which were Coiflets wavelets (Coif5) at level 14, the discrete cosine transform (DCT), and Daubechies wavelet (db4) at level 8. Three feature selection methods were compared, and PCA was considered as the best one for the application of the study as the recognition rate was constantly 100% for subject-dependent and subject-independent scenarios across the ADM as well as the DEM. The classification was conducted using PNN with a 0.01 sigma value. By far, this paper reports the highest claimed accuracy for a unimodal ECG-based emotion recognition system.

The work by Hovsepian et al. [117], for ECG classification of binary stress and non-stress (Pos/Neg), reported 89% and 95% accuracy, respectively. The classifier used was SVM with RBF kernels trained using HR, HRV, and non-HRV features. The raw ECG signals were filtered and normalized before being extracted. Validation was also conducted between subjects as more than twenty subjects participated in the study.

In a study by Selvaraj et al. [40], six classes of emotions from the ECG unimodal approach were successfully classified with a maximum accuracy of 92.87%. The experiment was conducted on sixty subjects by inducing happiness, sadness, fear, disgust, surprise, and neutral emotions. The features that were extracted from ECG signals were nonlinear features or Hurst features. The features were derived from RRS and FVS. They also proposed a novel Hurst feature by merging RRS and FVS with HOS. The dataset was separated with a ratio of 70:30 for training and testing datasets. Four classifiers were considered: Bayesian classifier, regression tree, KNN, and fuzzy KNN, where the last classifier performed the best.

Xun and Zheng [86] also managed to obtain 92% accuracy in classifying joy and pleasure from the AuBT dataset. They only utilized the ECG signals from the database to perform the study. The ECG features were extracted using AuBT toolboxes, which provided a combination of HR and HRV features. A total of 81 features were extracted, but only 5 final features were selected using a combination of analysis of variance (ANOVA), SFS, and SBS. The final selected features were *R_range*, *ecgRampl-std*, *ecgHrv-max*, *ecgHrv-range*, and *ecgHrvDistr-range*. The classification was conducted using SVM, LDA, and Fisher’s linear discriminant analysis with SVM as the best methods.

Guo [102] performed a comparison study between BPNN and RBFNN in classifying emotions using the AuBT dataset. The accuracy result for BPNN was 87.5%, while for RBFNN, it was 91.6%. The ECG features extracted were from the multiscale wavelet decomposition method for the extraction of the maximum value of wavelet coefficients and the standard deviation. The study highlighted that wavelet coefficients that are treated as eigenvectors are able to effectively characterize ECG signals.

Meanwhile, in Table 4, there are seven works that reported more than 90% accuracy in classifying emotions based on varying emotional models and multiple modalities inclusive of ECG. Lee and Yoo [109] collected multimodal physiological signals from ECG, EDA, and SKT from 15 subjects. The highest classification accuracy was found using NN at 92.5%, while 85.6% and 81.2% were found using QDA and LDA. The study also showed that a higher accuracy is expected by applying feature engineering through multimodal feature extraction and feature selection. The features extracted from ECG signals are time domain HRV features. The feature selection algorithm used was Kullback–Leibler divergence. EDA features were selected more frequently than the others, but as for ECG features, RMSSD, NN50, SDNN, and LF/HF were among the selected features in subject-dependent scenarios. The affective model used was Pos/Neg as the collected samples were based on fear as the negative label, and normal as neutral.

In [100], Gong et al. managed to classify joy and anger with 100% accuracy, while pleasure and sadness were classified at 92% and 88%. The study was conducted using the AuBT database and utilized a multimodal approach. The ECG, EMG, SC, and RSP were extracted using the ensemble empirical mode decomposition (EEMD) method, and the classifier used was C4.5 DT.

The authors of [115] focused on the combination of ECG and EEG for the application of an emotion recognition interface for interactive contents. The feature extracted from the ECG signals was HRV, and the classifiers tested were MLP, SVM, and a Bayesian network. By adopting 10-fold cross-validation, the best classifier reported was the Bayesian network, with 98.06% accuracy in recognizing six emotions from the DEM. Collected from 30 subjects, the emotions were amusement, fear, sadness, joy, anger, and disgust.

Kim and Andre [69] collected ECG, EMG, SC, and RSP signals from three subjects and performed a feature-based multiclass classification. The ECG features extracted were based on the HRV time, frequency, and nonlinear domains. Using a novel technique called emotion-specific multilevel dichotomous classification (EMDC), the authors managed to obtain a 95% average accuracy for subject-dependent and 70% for subject-independent scenarios. Among 110 combined extracted features, the best emotion-relevant feature from ECG was SD2 from the HRV Poincare plot for valence, arousal, and four classes of valence/arousal.

The study by Wagner et al. [85] adopted the AuBT multimodal physiological signal approach for emotion recognition. The ECG features extracted were HR statistical values. A few feature selection and classification techniques were tested to assess the recognition performance. With 92.05% accuracy, the four classes of emotion were classified using the linear discriminant function (LDF), and the features were selected using SFS. The same configuration obtained 96.59% accuracy on classifying arousal. However, for valence, the highest accuracy achieved was 88.64% using MLP and the combination of Fisher and SFS.

Healey and Picard [52] performed emotion recognition through detecting stress in a real-world driving scenario. A total of 24 drivers were tested through different traffic conditions in the greater Boston area while continuously providing feedback on their stress level. ECG, EMG, SC, and RSP sensors were attached to their body, and the data were recorded. The ECG features extracted were from the HRV power spectrum and sympatho-vagal balance ratio. The Fisher projection matrix and linear discriminant were used to determine the accuracy of the Pos/Neg emotional model. High, medium, and low stress recognition accuracies were 97.4%, 94.7%, and 100%, respectively.

Lastly, Haag et al. [78] took a multimodal approach towards emotion recognition by incorporating ECG, EMG, EDA, ST, RSP, and BVP. The ECG features extracted were HR, HRV, and IBI. Using NN, the study managed to classify arousal with 96.58% accuracy, and valence with 89.93% accuracy.

## 7. Application of Emotion Recognition System in Healthcare

A lot of treatments are available for physical illness, but it is not the same for psychological illness. Emotional health is important for the wellbeing of one’s mental state. A negative emotional state may cause social and physical problems if left undiagnosed and untreated. For instance, prolonged exposure to stress or depression may lead someone to withdraw from a healthy relationship with the people around them and being aggressive, which could be dangerous for him/herself and the people around them. Moreover, negative emotions may also cause physical problems such as headaches, stomach upset, and muscle ache. An emotion recognition system can be utilized to improve the healthcare sector, especially in addressing metal health issues.

### 7.1. Emotion Recognition Application in Healthcare Utilizing ECG

The authors of [7,18] proposed a new healthcare system that focuses on emotional wellbeing. The system consists of physiological sensors (ECG and EEG) to measure and detect emotions. Based on that, the system provides necessary services such as relaxation, amusement, and excitement. These three emotional services are selected to balance out negative emotions detected from the subject with strong positive states. The relaxation service consists of a guided deep breathing exercise proven to benefit stress management. The exercise came with virtual objects in augmented reality and musical assistance for a calming effect. The system utilizes augmented reality as an output service channel, thus providing amusement and excitement services to the user interaction with the virtual objects. The interaction is enabled by Kinect’s gesture detection.

A healthy workplace environment using a novel mood recognition solution that is able to identify eight different DEM emotions in every two-hour interval was proposed in [105]. The employees were provided with a wearable physiological device (ECG, PPG, and TEMP) along with a complimentary smartphone application called “HealthyOffice”. The configuration setup was conducted to facilitate a periodical self-reporting towards the current emotional state in a structured manner. The objective of constantly monitoring employees’ emotions in the workplace is to optimize the overall mental health of the organisation by eliminating anxiety, stress, and depression in the working environment. Thus, higher productivity is expected, and the output revenue can be significantly measured. A similar study of emotion healthcare application in the workplace environment was also conducted in [77], with a slightly different approach. This study used ECG, EDA, and TEMP as the physiological models. Rather than identifying the spectrum of basic emotions, the work only focused on stress and non-stress binary emotional classification.

A clinical application of emotion recognition systems was presented by [117]. The study utilized ECG and respiration sensors to detect stress symptoms in the patients. The targeted application of the work was towards patients who suffer migraine, addiction (substance or smoking), and stress-related disorders. The benefit of monitoring the patients’ emotional stress condition is to ensure that a negative tendency is not triggered. Daily stress management can reduce severe addictive behavior and refrain from triggering migraine. The work also proposed a combination of physiological signals and other data such as visual exposure, social interactions, geoexposures, light and sound exposures, and digital trails to determine which parameters influence stress triggers.

In [119], a home healthcare system using wearable physiological sensors that have an emotion recognition function was designed. The targeted groups for the application of the system were elderly and sub-healthy people. HR, TEMP, and SC were monitored at the wrist of the wearer in real time. The data were broadcast wirelessly to the family doctor or health practitioner who is responsible for the subject. An alert system was also embedded in the design to send a text message and notify the doctor, in case of a risky situation. The healthcare system can detect the states of joy, anger, and sadness.

The cardiac defense response (CDR) is a specific field of study that is closely related to psychophysiological reactivity towards an intense stimulation. CDR serves as a protective function of the fight or flight response in case of dangerous situations [120]. However, when exposed to it for a long period of time, anxiety, stress, depression, and other mental disorders might arise. The author of [121] proposed a novel integrated system using ECG signals to detect fear in real time. Since fear is the emotional response when a person is in danger, the system was designed to detect a prolonged CDR. In healthcare, this system is important for monitoring stress and early prevention of mental disorders.

### 7.2. General Healthcare Application of Emotion Recogntion Systems

The application of emotion recognition in military healthcare was studied in [122]. Since armed forces are constantly exposed to a highly stressful scenario and environment, many of them tend to develop psychiatric conditions such as depression, post-traumatic stress disorder (PTSD), and suicidal thoughts. To prevent dispatching emotionally unstable personnel into a risky mission, the work proposed the usage of emotion recognition screening to assess the mental health status of the subject. The system also analyzed the reaction towards stressful emotions of the subjects. However, further development is still needed for any practical application.

Next, an emotion recognition system was applied in [123] to improve the patient e-healthcare system in a so-called smart city. Medical doctors have difficulties in detecting and controlling the degree of pain experienced by their patients, especially for patients who cannot express it verbally such as babies. Thus, the study proposed a remote patient monitoring system that employs an automatic emotion detection architecture. The system is capable of achieving a more personalized pain detection index through emotion monitoring. With a proper analysis provided, the result of this system manages to obtain an accuracy of approximately 90% using SVM as the classifier.

Faiyaz et al. [124] proposed a novel e-healthcare support system with emotion recognition using fuzzy logic. The framework designed is suitable in the context of a real-life healthcare environment. Monitoring patients’ emotions through the e-health system influences their satisfaction, wellbeing, and physical health. With the emotional feedback from their customers, healthcare providers can improve the quality of their services. The way of treating with empathy can be instilled in medical practitioners when they are aware of the affective state of their patients. This system is beneficial to both parties and improves the overall standards of the healthcare industry.

A fairly recent study was conducted in detecting the emotional state of patients during the spread of the virus SARS-COV-2, where face masks are mandatory [125]. A facial emotion recognition study was conducted with masked and unmasked versions of data. The unmasked faces in the database were modified digitally to add an artificial blue surgical mask over the face of the subjects. The system was designed to encourage pleasantness in doctor–patient interaction. However, with face masks being worn, inter-professional communication in healthcare is being upheld by the adoption of emotion recognition systems.

Another study that used computer vision to detect emotions in a healthcare center was presented in [126]. A multimodal visualization analysis was conducted on the facial expression of patients monitored using a monitoring camera at different intervals. The data were transmitted using the Internet of Things (IoT) and processed at the analysis center. If the system detected an abnormal expression, it would alert the physician in charge to check up on the patient.

Mental disorders and depression are serious illnesses that reduce the quality of life of individuals and the people around them. Early diagnosis of these psychiatric diseases can be conducted using an emotion recognition system, as proposed in [127]. The psychiatric patient-centric pervasive (P-cube) platform was designed to connect with the subject’s smartphone or laptop to collect data for emotion recognition. Utilizing speech data recorded from the headset, the system can provide the therapist with deeper affective insights into a subject’s mental state. Six basic emotions are detected using the system: anger, boredom, desperation, disgust, happiness, and pride.

Finally, ref. [128] proposed a speech signal-based emotion recognition system to analyze and detect compounded emotions. Prolonged anger, fear, and sadness are compounded with anxiety, where the person is prone to develop a more serious mental and physical health condition in the future. Compounded emotions might also drive a person to use substances, and, in the worst case, to commit suicide. The study designed a neural network-based autoencoder to extract suprasegmental features in voices and detect the early symptoms of anxiety disorder.

## 8. Discussion

### 8.1. Summary of the Review

The objective of this work was to perform a comprehensive review on emotion recognition systems that adopt ECG signals, and on their applications in healthcare. From the research reviewed, it is shown that with a combination of good pre-processing techniques, feature extraction and selection methods, and classification algorithms, human emotions can be recognized by machines with a medium to good accuracy. Even though the research on affective computing has been around for more than a decade, a standard universal emotional model has still not been achieved. Emotional models such as the ADM, DEM, and Pos/Neg are still ambiguous, particularly in the number of classes for the DEM. There are three-class, four-class, and even five-class labels for the DEM, which somehow raise the question of the purpose of recognizing each emotion. However, with the valence and arousal scale in the ADM, and the stress and non-stress binarization of Pos/Neg, the targeted application of emotion recognition systems is more focused and simpler.

The other angle reviewed here is how extracted ECG features are relevant to the ANS activity in the heart. Our eyes cannot visibly capture any characteristic changes in the raw ECG signal; however, the feature extraction techniques are sensitive enough to extract the informative features of ECG. Additionally, feature selection and dimensionality reduction allow only the most relevant features to be adopted to recognize the specific emotion, while features that are unnecessary are eliminated.

The classification and validation steps are the most important parts in emotion recognition systems. Different classifiers use different learning approaches towards the data being trained. Even though the most used machine learning algorithm for emotion recognition systems is SVM, it is not necessarily the best approach. As it was previously discussed, there are few studies that managed to outperform SVM’s performance with other machine learning models. In addition, the reason most research on emotion recognition used machine learning instead of deep learning is because of the scarcity of the data available. As it was summarized, in the available databases, the number of subjects and samples are less compared to medical databases that deal with cardiac disease. Nonetheless, deep learning has been considered and has shown a promising performance. With more data, deep learning is a good direction for this area. However, collecting a large database to perform a subject-dependent and subject-independent analysis requires a lot of time and cost. Thus, it is important for researchers to properly decide the pipeline of their research and consider validation techniques in order to increase variability.

Finally, application of emotion recognition systems in healthcare focusing on mental health was reviewed in Section 6. Emotion recognition systems are able to help in assessing the mental state of an individual. The output of the system can then be used as an input for a system that responds to the emotion to provide comfort and regulate the emotion so that a positive emotion is experienced by the individual.

### 8.2. Research Challanges

Among the studies reviewed, the challenge for ECG-based emotion recognition systems is the lack of affective databases with a large number of samples taken from subjects with different backgrounds. Current affective databases are limited by an age group bias, where only university students participated in the data collection processes. Moreover, one of the regional experiments conducted caused the database used to have a homogenous locality sample from people with the same ethnic backgrounds.

The next challenge comes from the perspective of annotation, as well as unstandardized emotional models and scales. Since emotions are subjective experiences defined through different perspectives, the inexactness may cause classification fallacies. If the emotion experienced by a subject contradicts the perceived emotions by a second- or third-person perspective, this might cause a huge mess in the system. When dealing with insufficient datasets, researchers tend to combine datasets to increase the sample size. The unstandardized emotional models and scales cause a huge challenge in adopting different affective datasets in one study.

The last challenge is the applicability of emotion recognition systems designed for real-world situations, especially in healthcare. The majority of the studies summarized are not available for actual use because of the complexity of the design. The whole purpose of academic research is to promote intelligent solutions to issues or problems faced in real life. However, since the studies are not repeatable or are difficult to replicate, other researchers have difficulties in improving the steps taken from previous works. In order to make emotion recognition systems common in the healthcare industry, the models proposed have to be simple, efficient, and reliable, in addition to being tested vigorously.

### 8.3. Future Works

Further research should be conducted on emotion recognition systems based on ECG signals for healthcare purposes. Primarily, the relationship of different age groups, ethnicities, and personalities towards emotion stimuli and responses should be investigated. The bigger the sample size with a heterogenous background, the better the classification approach, and thus a universal system can be built. Next, the perspective of intercompatibility between one dataset and another should be reviewed if the same methodologies are to be applied to compensate the training and testing accuracy and promote the generalizability of the developed system. The research of emotion recognition should be closer to a real-life scenario, where the computer can learn to eliminate more outside noise, instead of working in a controlled environment. By applying this approach, the system should be robust and versatile for further application and commercialization. By deploying emotion recognition systems for healthcare usage, the architecture built must be reliable in dealing with different scenarios. Finally, various other possible real-world use cases of emotion recognition systems which allow personalization in real time should be explored.

## 9. Conclusions

This review has shown that emotion recognition systems are an essential subject in healthcare, and the application of them is possible via ECG as a unimodal or multimodal approach. The growing trend of research related to emotion recognition systems is a heathy step towards the maturity of this field. Future endeavours of incorporating emotional health in technological development will contribute to more responsible and sustainable innovations.

## Figures and Tables

**Table 1:** Number of papers reviewed from the respective databases.

Publisher Database	ECG and Emotion Recognition	Healthcare Application
IEEE Xplore	26	9
Science Direct: Elsevier	4	1
Nature: Scientific Reports/Data	4	0
ACM DL	3	1
Springer Link	3	2
MDPI	2	0
IOP Science	2	1
J-Stage	1	0
Springer Nature	1	0
IOS Press	1	0
Wiley Online Library	1	0
Fuji Technology Press Ltd.	1	0
IJEECS	1	0
Frontiers	1	0
**Total**	**51**	**14**

**Table 2:** Affective datasets available with the inclusion of the ECG modality.

Dataset	Stimuli	Data Size (Participants × Samples)	Physiological Modalities	ECG Device	ECG LEAD	Sampling Rate	Emotional Annotations	Emotional Model	Annotation Perspectives
AMIGOS [55]	51–150 s videos	40 × 16	ECG, EEG, GSR	Shimmer	RA-LL (Lead 2), LA-LL (Lead 3)	256 Hz	Valence, Arousal, and Dominance	ADM	Self and 3rd person
ASCERTAIN [56]	51–128 s videos	58 × 36	ECG, EEG, GSR	NA	2 Leads	256 Hz	Valence and Arousal	ADM	Self
AuBT [66]	2 min of music listening	1 × 100	ECG, EMG, RSP, SC	NA	1 Lead	256 Hz	Joy, Anger, Sadness, and Pleasure	DEM	Self
CASE [67]	<3 min videos	30 × 20 (real time)	ECG, BVP, EMG, GSR (EDA)	Thought Technology	RA-LA (Lead 1), RA-LL (Lead 2), LA-LL (Lead 3)	1000 Hz	Valence and Arousal	ADM	Self
CLAS [68]	16 video and 16 IAPS pictures	62 × 32	ECG, PPG, EDA	Shimmer3	RA-LA (Lead 1)	256 Hz	Valance and Arousal	ADM	Self
DECAF [57]	1 min music videos and ~ 80 s movie clips	30 × 76	ECG, EMG, EOG, MEG	NA	RA-LA (Lead 1)	1 KHz downsampled to 256 Hz	Valence, Arousal, and Dominance	ADM	Self
DREAMER [58]	65–393 s film clips	23 × 18	ECG, EEG	Shimmer ECG	RA-LL (Lead 2), LA-LL (Lead 3)	256 Hz	Valence, Arousal, and Dominance	ADM	Self
DSDRWDT [52]	50–90 min of driving	24	ECG, EMG, SC, RSP	FlexComp	RA-LL (Lead 2)	496 Hz	Low, Medium, and High Stress	Pos/Neg	Self
EMDC [69]	3–5 min of music listening	3 × 360	ECG, EMG, SC, RSP	Procomp2 Infiniti	3 Leads	256 Hz	Valance and Arousal	ADM	Self
K-EmoCon [51]	10 min naturalistic conversations	32 (real time)	ECG, EEG, BVP, EDA, SKT	Polar H7		1 Hz	Valence and Arousal	ADM	Self, 2nd and 3rd person
MANHOB-HCI [59]	35–117 s videos	27 × 20	ECG, EEG, GSR, EDA, RSP, SKT	Biosemi Active II	3 Leads	1024 Hz downsampled to 256 Hz	Valence, Arousal, and Dominance	ADM	Self
MPED [31]	<5 min videos	23 × 28	ECG, EEG, GSR, RSP	Biopac System	3 Leads	250 Hz	Joy, Funny, Anger, Fear, Disgust, Sad, and Neutral	DEM	Self
SWELL [70]	Writing, presenting, reading, and searching	25 × 3	ECG, SC	Mobi device (TMSi)	3 leads	2048 Hz	Valance, Arousal, and Stress	ADM and Pos/Neg	Self
WESAD [71]	Video clips, and public speaking and mental arithmetic tasks	15	ECG, BVP, EDA, EMG, RSP, TEMP	RespiBAN Professional	3 Leads	700 Hz	Neutral, Stress, and Amusement	Pos/Neg	Self

**Table 3:** Research that only uses ECG as a unimodal approach or a multimodal physiological approach, with ECG standalone accuracy results included.

Source	Dataset	Modalities	ECG Pre-processing	ECG Extracted Features	Features Selection	Selected Features	Classifier	Validation	Accuracy
[20]	Own: 11 subjects, 56 music listening DEM (happiness, sadness, peacefulness, scary, neutral) and ADM (valence, arousal)	ECG, GSR	Digital notch filter at 50 Hz	Matching pursuit. Min, mean, max, var, std of Coif5 at level 14, db4 at level 8, DCT	LDA, PCA, Kernel-PCA	NA	PNN	NA	Subject-dependent and subject-independent using PCA: 100%
[21]	Own: 44 subjects, 5 images ADM (valence, active/passive arousal)	ECG	NA	EMD (bivariate extension of EMD), Hilbert–Huang Transform, local oscillation in every mode	NA	NA	LDA	NA	up to 89%
[24]	Own: 12 subjects, 60 samples each DEM (anger, fear, sadness, disgust, joy, neutral)	ECG	Interpolation filters remove 30 Hz and below	- IBI - WIB	Least Significant Difference—ANOVA	36 features: 11-feature approach and 3-feature approach	LDA, Adaptable KNN	NA	11-feature approach 37.23% 3-feature approach 61.44%
[26]	Own: 25 subjects, 488 samples DEM (anger, sadness, joy, pleasure)	ECG	Butterworth bandpass filter 0.05–100 Hz	- PQRST, HRV: sdnn, mn_nn, rmssd, m_nn, nn50, pnn50, hf, hfnu, lf_hf, lfnu, total_power, vlf, sd1, sd2 - WIB: PR, ST, QRS min, max, std, mean, med - EMD: spectral power of IMF in time and frequency domain, instantaneous frequency of IMF, spectral power of instantaneous frequency of IMF - TFB (ten-frequency band)	Recursive Feature Elimination, Chi-Square Test, P test, RF FS, Extra Tree FS, Random SVM FS	- EMD: spec_2, spec_4 - HRV: sdnn, mn_nn, m_nn - WIB: median_pr, max_pr, sd_pr, mean_qrs, max_qrs, min_qrs - TFB: band_2, band_3, band_5, band_7, band_10	RF, Extra Tree, Gradient Boost, AB SVM, AB DT, AB Naïve Bayes	10-fold CV	80% extra tree classifier and feature selection 79.23% RF classifier and extra tree feature selection 72.66% gradient boost classifier and RF FS
[39]	Own: 5 subjects, 15 video clips (Pos, Neg, Neutral)	ECG	Elliptic bandpass filter, DWT	Time domain: HR, MRAmp, MRRI	NA	NA	KNN, SVM	10-fold CV	Pos/Neg Neutral KNN: 66.49% 60:40 train/test, 66.22% 70:30 train/test, 67.54% 80:20 train/test Pos/Neg KNN: 74.67% 60:40 train/test, 77.69% 70:30 train/test, 77.42% 80:20 train/test Pos/Neg SVM: 64.98% 60:40 train/test, 65.52% 70:30 train/test, 66.04% 80:20 train/test
[40]	Own: 60 subjects, 60 samples DEM (happiness, sadness, fear, surprise, disgust, anger)	ECG	Baseline wander removed using wavelet-based algorithm, 6th-order Butterworth filter with 45 Hz cut-off	Nonlinear features “Hurst” using RRS and FVS from QRS. Combined HOS: Hurst, skewness based on Hurst, kurtosis based on Hurst	NA	NA	Bayesian classifier, Regression tree, KNN, Fuzzy KNN	Random validation, Subject-independent validation	Fuzzy KNN 6 Class: 92.87% RRS, 76.45% FVS
[47]	Own: 8 subjects DEM (joy, anger, sadness) + AuBT	ECG	2nd-order IIR notch filter, Butterworth low-pass filter with 60 Hz cut-off frequency	LBP, LTP: 3 s, 5 s, 10 s, 15 s frame length, and 1.5 s, 2.5 s, 5 s, 7.5 s frame shift	NA	NA	KNN	10-fold CV	LBP 84.17%, LTP 87.92%
[55]	AMIGOS	ECG, EEG, GSR	NA	Root mean square of IBI, mean IBI, 60 spectral power, LF, MF, HF of HRV spectral power, HR, HRV statistics: mean, std, skewness, kurtosis, % of time the future value above/below mean ± std	PCA	NA	Linear SVM	10-fold CV, LOOCV	Short video scenario: 53.5% V, 55.0% A Long video scenario: 55.0% V, 54.3% A Both: 54.5% V, 55.1% A
[56]	ASCERTAIN	ECG, EEG, GSR	NA	10 low-frequency PSD, 4 very slow response PSD, IBI, HR, HRV statistics: mean, std, skewness, kurtosis, % of time the future value above/below mean ± std	NA	NA	Linear SVM, NB	LOOCV	SVM: 56% V, 57% A NB: 60% V, 59% A
[58]	DREAMER	ECG, EEG	No pre-processing	- PQRST features: mean, med, std, min, max, range - HRV: RMMSD, PSD LF, PSD HF, LF/HF, total power	NA	NA	RBF SVM	NA	62.37% V, 62.37% A
[71]	WESAD	ECG, BVP, EDA, EMG, RSP, TEMP	NA	- HR, HRV: mean, std - HRV: NN50, pNN50, TINN, RMS, ULF, LF, HF, ULF, LF/HF, fULF-HF, relative power, normalized LF, normalized HF	NA	NA	DT, RF, AB, LDA, KNN	LOSO CV	3 Class: DT 57.81%, RF 60.36%, AB 61.71%, LDA 66.29%, KNN 54.76% Pos/Neg: DT 80.17%, RF 82.78%, AB 83.37%, LDA 85.44%, KNN 79.19%
[74]	Own: 16 subjects, 96 samples ADM (valence, arousal)	ECG	Butterworth low pass filter	HRV	Genetic Algorithm	NA	SVM	5-fold CV	ADM: 72.9% 89.6% V, 82.3% A
[75]	Own: 6 subjects, 36 film clips (Pos, Neg, Neutral)	ECG, EEG, RSP	Remove baseline drift, 1–60 Hz bandpass filter, 49–51 Hz band-stop Butterworth filter	HR, HR stability (HRstd), power (Hpow)	NA	NA	Linear SVM	NA	Pos/Neg, Neutral: HR 69.0%, HRstd 84.2%, Hpow 70.4%
[76]	Own: 30 subjects, 60 video clips DEM (happiness, sadness, fear, surprise, disgust, neutral)	ECG	4th-order notch filter at 50 Hz, 4th-order Butterworth filter 100 Hz cut-off, digital high-pass filters	EMD combined with Hilbert transform, EMD combined with DFT	NA	NA	LDA, KNN	NA	KNN: 52%
[77]	Own: 34 subjects, Pos/Neg (stress, no stress)	ECG, EDA, ST	5–15 Hz bandpass filter	HRV time and freq domains: mRR, medRR, mHR, SDRR, RMSSD, RR50, pRR50, LF, HF, LF/HF	Wrapper method	HRV: mRR, medRR, mHR, SDRR, RMSSD, RR50, LF, HF, LF/HF	LDA, QDA, SVM, KNN	LOOCV	KNN: 88.03%
[84]	Own: 391 subjects, 10 film clips (joy, sadness)	ECG	35 Hz low-pass filter and 50 Hz power source notch filter	CWT, 79 features: mean, std, med, min, max, range of intervals. P, R, S amp, HRV, and PSD	BPSO, HPSO	20, 16: Most selected: max R, range R, mean R, med R, range QS, std PQ, std S, mean QS, std QS, med P	Fisher classifier	Run 40 times	Joy: 84.45%, Sadness: 88.43%
[86]	AuBT DEM (only joy and pleasure data)	ECG	NA	81 features of HR and HRV	ANOVA: 44 features, SFS: 37 features, SBS: 3 features	R-range, Rampl-std, HRV-max, HRV-range, HRVDistr-range	SVM, LDA, Fisher’s linear discriminant	NA	SVM + SFS-SBS-ANOVA: 92%
[89]	DECAF	ECG	Butterworth filter	HR, DWT, QRS morphology: qrsWBR, qrsWRE, qrsABR, qrsARE, qrsMOR	NA	NA	SVM, RF	NA	63.4% RF 64.5% SVM
[99]	AuBT	ECG, EMG, SC, RSP	Adaptive low-pass filter	HHT (EMD and Hilbert transform) fission and fusion	NA	4, 8, 12, 16 IMF features	SVM	10-fold CV	Fission 69%, Fusion 56%
[101]	AuBT	ECG, EMG, RSP, SC	NA	16 features of CWT Morlet wavelet coefficients	NA	NA	SVM	NA	75%
[102]	AuBT	ECG	NA	Wavelet transform: max and std of multiscale wavelet coefficients	NA	NA	BPNN, RBFNN	NA	BPNN: 87.5%, RBFNN 91.67%
[103]	Own: 391 subjects, 10 film clips DEM (joy, sadness)	ECG	NA	DWT, 79 features	Tabu Search Algorithm	23, 12: Most selected: std S, max R, std QS, range R, mean S, med R, med S, std R, min R, min S, PNN50 HRV, LF HRV	KNN, Fisher-KNN	Run 9 times	KNN: 75.85%, Fisher-KNN: 85.78%
[104]	Own: 20 subjects, 400 samples DEM (happy, sad, pleasant, angry)	ECG	NA	- Statistical features of time and frequency domain: max, min, mean, std, rrmean, rrstd, energy, ratio - DFA: α, α1, α2 - Multifractal Features: α0, Δα - DCCA: ρDCCAh, ρDCCAm, ρDCCAl	Max–Min Ant System, Ant Colony Optimization	NA	KNN, SVM, DT	CV	Best Classifier: KNN 4 Class: 92% Happy 91% Sad 92% Pleasant 88% Angry 97%
[106]	Own: 30 subjects, 110 samples DEM (sadness, anger, happiness, relaxation) and ADM (valence, arousal)	ECG	Artifact removal and filtering	Instantaneous Spectrum and Bispectrum, Dominant Lyapunov Exponent	NA	NA	SVM	LOOCV	4 Class: 79.29% V/A: 79.15%, 83.55%
[108]	AMIGOS, DEAP, DREAMER, MANHOB-HCI	ECG, EEG, GSR, EDA, RSP, SKT, etc.	Moving average filter with 0.25 s window length	HRV: pNN50 Spectrogram: 4096 features	PCA	30 features	Extreme learning machine	NA	V/A (Individual): DEAP 70.86%, 71.09%; AMIGOS 81.89%, 82.74%; MANHOB-HCI 78.76%, 78.76%; DREAMER 80.43%, 80.68% (Combined): DEAP and AMIGOS 59.69%, 63.61%; DEAP, AMIGOS, and MANHOB-HCI 58.57%, 61.84% (Transfer Learning): Train (DEAP and AMIGOS) Test (MANHOB-HCI) 64.77%, 62.50%; Train (DEAP) Test MANHOB-HCI 63.59%, 61.46%
[111]	Own: 25 subjects, 50 samples each Pos/Neg and DEM (sad, angry, fear, happy, relax)	ECG	NA	- HRV: Time Domain (Mean RRI, CVRR, SDRR, SDSD) - Frequency Domain (LF, HF, LH ratio) - Statistic Analysis (Kurtosis coefficient, Skewness, Entropy) - Parameters of Poincare Plot (SD12, SD22, SD2SD1ratio)	PCA	Selected 5 from 13. CVRR, LF, HF, HF ratio, SD1	SVM	NA	Pos/Neg: 71.4% 5 Class: 56.9%
[112]	AMIGOS, DREAMER, WESAD, SWELL	ECG	High-pass IIR filter with bandpass of 0.8 Hz. Z-score normalization	High-level spatiotemporal features	NA	NA	Self-Supervised CNN	10-Fold CV	AMIGOS: 87.5% V, 88.9% A; DREAMER: 85.0% V, 85.9% A WESAD: 96.9% Pos/Neg; SWELL: 97.3% V, 96.7% A, 93.3% Stress
[114]	Own: 21 subjects, (Pos, Neg)	ECG, RIP	Tomkins’s algorithm	9 features: heartbeat freq low, med, high, ratio low/high; QD, SD, 3.32QD	Correlation-based feature selection	HR power in Bands 1 and 3, mean, med, and 80th percentile of stretch	SVM	10-fold CV	~85%
[113]	AMIGOS	ECG, GSR	Pan–Tompkins QRS detection. 0.5–15 Hz cut-off frequency removal	- IBI time domain: meanNN, medNN, SDNN, rmSSD, pNN50, pNN20, coefVarSD, medADNN, coefVarNN, mCoeffVarNN, Shanon Entropy, HRV triangular, numArtifacts - Freq domain: peakHF, hTotalPowerRatio, normHF, peakLF, lfhfRatio, lfTotalPowerRatio, normLF, totalPower, ulfPeak, vhfPeak, vlfPeak - Nonlinear domain: correlation dimension, entropy, SVD, HF, LF, VLF, Shannon, fractal dimension Higushi and Petrosian, Fisher information	NA	NA	DCNN	NA	71% V, 81% A
[116]	Own: 25 subjects, 3 movies DEM (fear, disgust, neutral)	ECG	Quantization, Normalize Relative Compression Measure	NA	NA	NA	1-NN	Leave-one-out strategy	Fear: 77%, Disgust: 63%, Neutral: 74%
[117]	Own: 26 subjects Pos/Neg (stressed, not stressed)	ECG, RSP	Filtered and normalized	- HRV: var, quartile deviation, low freq energy, med freq energy, high freq energy, low/high freq energy ratio - Non-HRV: mean, med, 80th percentile, 20th percentile - HR	NA	NA	RBF SVM	Cross-subject validation	95% Not stressed, 89% Stressed

**Table 4:** Multimodal research that includes ECG model but did not perform an independent classification for the signal.

Source	Dataset	Physiological Modalities	ECG Pre-Processing	ECG Extracted Features	Features Selection	Selected Features	Classifier	Validation	Accuracy
[6]	Own: 10 subjects (Pos/Neg)	ECG, EMG, RSP, EDA	Low-pass filters at 100 and 500 Hz	HR, mean amp, mean abs first difference	NA	NA	SVM, adaptive neuro-fuzzy inference system (ANFIS)	10-fold CV	SVM 79.3%, ANFIS 76.7%
[51]	K-EmoCon	ECG, EEG, BVP, EDA, SKT	NA	HR	NA	NA	NA	NA	NA
[52]	Own: 24 subjects, 112 samples (Pos/Neg)	ECG, EMG, SC, RSP	NA	HRV: power spectrum, LF, HF, LF/HF, sympathovagal balance ratio, MF	ANOVA	NAs	Fisher projection matrix, linear discriminant	NA	97%
[53]	Own: 58 subjects DEM (anger, boredom, fear, frustration, happiness) and ADM (valance, arousal)	ECG, EDA, EMG, RSP	Baseline removal, filtering	- HRV/IBI, HR time domain: mean, med, max, min, range, var, std, ave derivative, abs deviation, kurtosis, skewness. - HR freq domain: 3 frequency bands, 4 energy bands	Fisher’ linear discriminant	~ 8 selected ECG features out of 173 features	Linear SVM	10-fold CV	V/A: 58.5% 5 class: 63.4%
[54]	Own: 30 subjects, virtual reality DEM (disgust, fear happy, sad)	ECG, PPG	EMD	IMF1, IMF2; 35 features: mean, max, std, min, log energy, var, skewness, kurtosis, rms, crest factor, shape fac, impulse fac, margin fac, energy, med, mean freq, rom of square level, band power occupied bandwidth, change points, power bandwidth, Shannon energy, mad, third-order interception, interquartile range, spurious free dynamic range, peak to rms, snd, thd, total jitter, ave freq, entropy	NA	NA	Ensemble bagged trees	5-fold, 10-fold, 15-fold CV	85.7%
[57]	DECAF	ECG, EMG, EOG, MEG	NA	IBI, HR, HRV, PSD	NA	NA	Linear SVM	NA	60% V, 57% A
[59]	MANHOB-HCI	ECG, EEG, GSR, EDA, RSP, SKT	NA	HRV, RMS of MSDFSB, SD, 56 spectral power, LF, MF, HF, HRV PS, Poincare analysis	NA	NA	RBF SVM	NA	(ECG + Peripherals): 45.5% V, 46.2% A
[67]	CASE	ECG, BVP, GSR, RSP, ST, EMG	NA	TEAP, Pan–Tompkins QRS detector: HR, IBI, SDNN	PCA	Mean HR	NA	MANOVA	NA
[68]	CLAS	ECG, PPG, EDA	NA	NA	NA	NA	Polynomial SVM	Leave one out	(ECG + PPG): V/A: ~70%
[69]	Own: 3 subjects, 120 samples ADM (valence, arousal)	ECG, EMG, SC, RSP	NA	FFT, SSE: meanEnergy_SubSpectra, meanHR_HRVtime, powerLow_HRVspec, mean_MSE, mean_SSE, etc.	NA	Valence 71, Arousal 45, 4 Class 77	SBS pLDA, EMDC	LOOCV	EMDC: Subject-dependent average: 95% Subject-independent: 70%
[78]	Own: 20 samples each ADM (valence, arousal)	ECG, EMG, EDA, ST, BVP, RSP	Low-pass filter with 90 Hz, sharp high-pass 0.5 Hz, notch filter 50 Hz	HR, HRV, IBI	NA	NA	NN	NA	89.93% V, 96.58% A
[107]	Own: 101 subjects, 4 video clips DEM (amusement, anger, grief, fear)	ECG, GSR, OXY	0.5 Hz high-pass filter, 35 Hz low-pass filter	Multi-variant correlation methods	NA	NA	RF	NA	74%
[85]	AuBT	ECG, EMG, SC, RSP	Low-pass filter, normalization	HR statistical values	ANOVA, SFS, SBS, PCA	NA	KNN, LDF, MLP	NA	4 Class: LDF-SFS 92.05% V/A: MLP-SFS-Fisher 88.64%, LDF-SFS 96.59%
[88]	Own: 22 subjects Pos/Neg (stress, stress-free)	ECG, EEG	NA	7 features: HR, HRV: VLF, LF, HF, LFnu, HFnu, LF/HF power ratio	Paired t-test, PCA		RBF and sigmoid SVM	10-fold CV	Sigmoid SVM 79.54%, RBF SVM 63.63%
[100]	AuBT	ECG, EMG, SC, RSP	NA	EEMD: Time Domain, Time Frequency Features, Nonlinear Features, IMF	NA	NA	C4.5 DT	NA	Joy 100%, Anger 100%, Sadness 88%, Pleasure 92%
[105]	Own: 4 subjects, DEM (excited, happy, calm, tired, bored, sad, stressed, angry)	ECG, PPG, ST	NA	HRVAS Toolbox (HAR, PWTT): IBI, SDNN, RMSSD, pNN50, HRVi, TINN; PDS Welch, Lomb-Scargle periodogram, Autoregression: VLF, LF, HF, normLF, normHF, LF/HF; Nonlinear: sampen, DFA, Poincare plot SD1, SD2, SD1/SD2	NA	NA	KNN, DT, Bagged Ensembled (BE)-DT, Personalized-Baseline, BLD	LO-participant-OCV	Best classifier: BE-DT: Personalized 70.60%, Generalized BE-DT 62.14%
[109]	Own: 15 subjects Pos/Neg (Fear, Normal)	ECG, ST, EDA	NA	HRV time domain: mean, SDNN, RMSSD, NN50, pNN50	Kullback–Leibler Divergence	Mixed	NN, LDA, QDA	LOOCV	NN 92.5%, LDA 81.2%, QDA 85.6%
[110]	Own: 14 subjects, 10 samples DEM (sadness, disgust, fear, happiness, neutral)	ECG, SC, RPS, ST	NA	HRV: - Time domain (SDNN, RMSSD, SDSD, pNN50, pNN20, FF) - Frequency domain (LF, HF, normLF, normHF, LF/HF)	SFFS, SFFS-FP, mRMR, mRMR-FP, ReliefF, ReliefF-FP, IG, IG-FP, OneR, OneR-FP, Chi2, Chi2-FP	NA	KNN, SVM, RF, ML, RIPPER, C4.5 DT, NB	LOSOCV	MLP 60.3%
[115]	Own: 30 subjects DEM (amusement, fear, sad, joy, anger, disgust)	ECG, EEG	NA	HRV	NA	NA	MLP, SVM, Bayesian Network	10-fold CV	Bayesian Network 98.06%
[118]	Own: 47 subjects (Pos/Neg Neutral)	ECG, PPG	NA	- 11 statistical time domain: SDNN, NN50, pNN50, SDSD, RMSSD, SDRR, δx, Nδx, Υx, NΥx, STDDRRI, - HRV RRI using STFT (short-time Fourier transform): LF, HF, TP, LF/HF, HF/(LF + HF). - HRV Poincare plot: SD1, SD2, SD12, Area0, Area1, Area2, Area3, Area4	NA	NA	CNN	NA	75.4%

## Data Availability

Not applicable.
